# Overexpression of FAM83A Is Associated with Poor Prognosis of Lung Adenocarcinoma

**DOI:** 10.1155/2022/8767333

**Published:** 2022-10-05

**Authors:** Xin Liu, Meng Fu, Daqing Xia, Zimei Ji, Nana Hu, Zaijun Leng, Wang Xie, Yuan Fang, Junqiang Zhang

**Affiliations:** ^1^Bengbu Medical College, Bengbu, 233030 Anhui, China; ^2^Department of Respiratory and Critical Care Medicine, The First Affiliated Hospital of USTC, Division of Life Science and Medicine, University of Science and Technology of China, Hefei, Anhui 230001, China; ^3^Anhui Province Key Laboratory of Medical Physics and Technology, Institute of Health and Medical Technology, Hefei Institutes of Physical Science, Chinese Academy of Sciences, Hefei 230031, China; ^4^University of Science and Technology of China, Hefei 230026, China

## Abstract

Family with sequence similarity 83, member A (FAM83A) plays an essential and fundamental role in the proliferation, progression, and apoptosis of many malignant tumors, including lung cancer. This study aimed to determine the expression pattern of FAM83A in lung adenocarcinoma (LUAD) and its correlation with the prognosis of cancer and the survival of the patients. Bioinformatics analysis, immunohistochemistry, and Western blotting were used to explore and detect the expression of FAM83A in LUAD cells. The mechanism of FAM83A in proliferation and migration was examined. The correlation between FAM83A expression and survival rate was assessed by the Kaplan-Meier and Cox regression. FAM83A expression was elevated in LUAD tissues and was related to shorter overall survival (*P* < 0.05). A significant increase in FAM83A protein was observed in the LUAD tissue (*P* < 0.05). Compared with patients with early-stage tumors (stage I-II), those with advanced stage tumors (stage III-IV) had significantly higher FAM83A expression levels (*P* < 0.05). Downregulation of FAM83A led to a reduction in cell proliferation, a decrease in migration ability, and diminished epithelial-mesenchymal transition (EMT) in the lung cancer cell lines. Overexpression of FAM83A was associated with early lymph node metastasis and poor overall survival among LUAD patients. The findings indicated that FAM83A may play a critical role in promoting the LUAD progression and thus might serve as a novel prognostic marker in LUAD.

## 1. Introduction

Lung cancer is one of the most common forms of cancer worldwide and also the leading cause of cancer mortalities worldwide [1]. In the recent years, the rate of incidence and mortality has significantly increased with significant gender and geographic differences. This is due to diversity in lifestyles and socioeconomic development [2]. GLOBOCAN reported approximately 2.21 million new lung cancer cases (11.4% of the total new cancer cases) and 1.80 million deaths (18.0% of the total cancer deaths) worldwide in 2020 [3]. Despite many therapies for cancer, no satisfactory clinical results have been observed because of early metastasis of tumor. Therefore, the main concern is on targeted therapies in order to increase the rate of recovery of the disease [4]. Traditional chemotherapies as the cornerstone of therapy in the first line setting in an advanced stage of lung cancer. In the past decade, significant improvements in survival rate have been observed. These improvements are due to the development of targeted therapies, i.e., epidermal growth factor receptor tyrosine kinase inhibitors (EGFR-TKIs), for lung adenocarcinoma through specific driver genes. Previous studies have demonstrated better therapeutic outcomes and fewer toxic effects via EGFR-TKIs compared with the traditional chemotherapies in patients with non-small cell lung cancer (NSCLC) and EGFR mutations [5–8]. However, resistance to EGFR-TKIs seems inevitable and limits its application in clinical practice. Despite the initial response, patients who were treated with third-generation EGFR-TKI Osimertinib would develop acquired resistance and disease progression occurred 9 to 18 months after treatment [9–11]. The patients' disease usually deteriorates rapidly following drug resistance.

FAM83A consists of 8 genes, FAM83A-H, and is a member of the FAM83 protein family which is located on chromosome 8q24 [12]. The FAM83 family of proteins contains a highly conserved DUF1699 domain at the N-terminal, which is thought to be closely related to the biological characteristics of the tumor [13]. Previous studies indicated the overexpression of FAM83A in a variety of tumors, such as lung and breast cancers, and suggested it as a potential biomarker for cancer prognosis and a therapeutic target [14]. FAM83A could be used to predict LUAD prognosis, while FAM83B could predict the prognosis of lung squamous cell carcinoma [15]. In addition to that, FAM83A (serine and protein rich) is correlated with the poor prognosis, in the case of lung adenocarcinoma. In EMT of lung cancer, FAM83A is also involved in the Wnt/*β*-catenin signaling pathway [16, 17]. The public databases of bioinformatics analysis demonstrated the possible role of FAM83A overexpressed in lung cancer. Several experimental studies have reported high expression levels of FAM83A mRNA in lung cancer tissue and circulating tumor cells [18, 19]. It is also evaluated by a bioinformatics analysis that there is correlation between the expression of FAM83A and programmed death-ligand-1(PD-L1) [20]. Shi et al. proposed that long non-coding antisense RNA FAM83A-AS1 could increase FAM83A expression and promote lung cancer cell growth [21]. However, most of the conducted studies mainly focused on detecting FAM83A expression at mRNA or antisense RNA levels, rather than on protein levels. Clear that increased mRNA expression does not always indicate high protein expression, while protein is the basic and ultimate biological functional unit of genes. Consequently, to clarify the biological significance of FAM83A in lung cancer, studies on protein levels rather than mRNA levels are required. In this regard, FAM83A expression at the protein level, the role of FAM83A in LUAD biological characteristics, and its effects on the clinical and pathological characteristics of LUAD patients were investigated in the present study.

## 2. Materials and Methods

### 2.1. Collection of LUAD Tissue Samples and Clinical Information

The tissue chip contained 84 paraffin-embedded LUAD and para-cancer tissue samples of lung cancer specimens surgically removed from patients. The surgical procedures were performed in The First Affiliated Hospital of the University of Science and Technology of China (USTC) from October 2004 to August 2008. The correlation of clinical and pathological features with FAM83A expression is shown in Table 1. All experimental protocols were approved by the Institutional Research Ethics Committee of The First Affiliated Hospital of USTC (No. 2019-P-017).

### 2.2. Immunohistochemistry

The expression of FAM83A protein in the lung tissue was assessed by immunohistochemistry. The tissue sections were deparaffinized and rehydrated by a LEICA Autostainer (Leica ST5010, Autostainer XL, Germany) at room temperature. Antigen retrieval (AR) was performed with the citric acid solution. The tissue pieces were washed and covered with rabbit anti-FAM83A antibody (1 : 400; No: orb183622, Biorbyt, Cambridge, UK) and incubated overnight at 4°C. Then, the slides were washed with phosphate buffer saline (PBS) and incubated with secondary antibody (Envision+/HRP, Rabbit, Dako, Sweden) and diaminobenzidene (DAB) solution for 30 and 5 min at room temperature, respectively, and counterstained with hematoxylin. The stained slides were examined and quantitatively analyzed using Image J, and average optical density (AOD) was evaluated. The AOD median value (MAOD) of the detected LUAD samples was calculated. The specimen with AOD ≥ MAOD and AOD < MAOD were defined as high and low expression, respectively [17].

### 2.3. Cell Culture

A549, H1395, H1795, and Calu-3 cells were purchased from the American Tissue Tradition Collection (ATCC) (Manassas, VA, USA). The cells were cultured in Roswell Park Memorial Institute (RPMI) 1640 medium (HyClone, Logan, UT, Aldrich, St. Louis, MO) at 37°C in a humidified incubator with a 5% CO_2_ atmosphere. The cells in the logarithmic growth phase (full to 70%-90%) were selected, shaken, and cleaned with 2 mL PBS, and then digested with trypsin at 37°C for about 1 min. The digestion was stopped by adding a complete medium, and the cell precipitation was used in subsequent experiments.

### 2.4. Western Blotting Assay

Western blotting was used to detect FAM83A and EMT-related protein expression in the cells. Standard procedures were performed according to the manufacturer's instructions. Briefly, proteins in cell lysates were separated by sodium dodecyl sulfate-polyacrylamide gel electrophoresis (SDS-PAGE) and subsequently transferred onto polyvinylidene difluoride (PVDF) membranes (Millipore, Massachusetts, USA) and incubated with the following primary antibodies against FAM83A (1 : 1000; Sigma, Saint Louis, MO, USA), *β*-Actin (1 : 10000; Abcam, Cambridge, MA, USA), vimentin (1 : 1000; Cell Signaling Technology, Danvers, MA, USA), E-cadherin (1 : 1000; Cell Signaling Technology, Danvers, MA, USA), and Snail (1 : 1000 Cell Signaling Technology, Danvers, MA, USA). The *β*-actin was used as a loading control [17].

### 2.5. Lentivirus Transduction and Generation of Stable Cell Lines

An HIV-1-based, lentiviral expression vector designed to express a small hairpin RNA (pLVX-shRNA1) was used for cell transduction. The small hairpin RNA (shRNA) oligonucleotide sequences targeting human FAM83A gene mRNA was designed and synthesized by Huada Gene Scientific and Technological Co., Ltd. (Shenzhen, China). The target sequence of FAM83A was 5′-CCGGAGGAAATTCGCTGGCCAAATCTTCAAGAGAGATTTGGCCAGCGAATTTCCTTTTTTT-3′. The lentivirus with shFAM83A-gene was produced by co-transfection of 293T cells and transfected into A549 and H1795 cells. Control cells were transfected with an empty vector. Stable cells were selected with puromycin (Beyotime, Nanjing, China) after infection. Positive clones were selected for further analysis [17].

### 2.6. Real-Time PCR (RT-PCR)

Total RNA was extracted from cultured cells using the E.Z.N.A Total RNA Kit (R6834-02, Omega, US). Reverse transcription was carried out using Prime Script RT Reagent Kit (Takara, Japan) according to the manufacturer's protocol. Reverse transcription polymerase chain reaction (RT-PCR) was then performed using SYBR Premix Ex Taq TM II Perfect Real-Time (DRR081A, TaKaRa, Japan) in Eppendorf Realplex 2S (Eppendorf, Germany). All primers were synthesized by Suzhou Jinweizhi Biotechnology Co., Ltd. (Suzhou, China). The primer sequences are as follows: FAM83A-F: 5′- CCAGACCGTCAAGCACAACA-3′, FAM83A-R: 5′-GGAGCACACAAACGAACACC-3′ [17].

### 2.7. Cell Proliferation Assay

Cell viability was assessed by a cell proliferation assay using cell counting kit-8 (CCK-8, Dojindo, Kumomoto, Japan). Briefly, the cell suspension was cultured in 96-well plates at a density of 1 × 104 cells per well. They were detected at 0, 12, 24, 48, and 72 h following the protocol. Cell growth rates were determined by measuring absorbance at 450 nm [17].

### 2.8. Wound-Healing Assay

Cells were cultured in a 6-well plate at a density of 1 × 10^6^ cells per well overnight. A wound was created using a 10 *μ*L pipette tip across the center of the well. After scratching, the wells were washed three times with PBS and incubated in a CO_2_ incubator at 37°C. Images were obtained immediately and 12 h after wounding. The healing of the wound surface areas was calculated and analyzed using the Image J tool (The healing areas =0-h areas – 24-h areas) [17].

### 2.9. Transwell Cell Assay

The cell invasion assays were performed using a Matrigel invasion chamber (pore size: 8 mm, BD Biosciences, USA) at a density of 1 × 10^6^/ml. Cells in serum-free medium were plated in the upper chamber. The chemoattractant in the lower chamber was 10% fetal bovine serum. After a 24-h incubation, the invaded cells were fixed with paraformaldehyde (PFA) and then stained with crystal violet. Finally, invaded cells were observed under an inverted microscope (Leica DMI 4000 B, Leica, Wetzlar, Germany) and manually quantified.

### 2.10. Bioinformatics Analysis

The study data and clinical information were provided from The Cancer Genome Atlas (TCGA) database. The University of Alabama at Birmingham cancer (UALCAN) data analysis portal (http://ualcan.path.uab.edu/analysis.html) was used to analyze the data. The Kaplan-Meier analysis was used to determine the relationship between FAM83A expression and the prognosis of the disease.

### 2.11. Statistical Analysis

Data was analyzed by SPSS 13.0 (Chicago, IL, USA). Correlation analysis was performed using Pearson's chi-square test. Prognostic factor analyses were performed using univariate and multivariate Cox regression analysis. *P* < 0.05 value was considered statistically significant.

## 3. Results

### 3.1. Upregulation of FAM83A in LUAD by Bioinformatics/UALCAN Analysis

**Figure 1:**
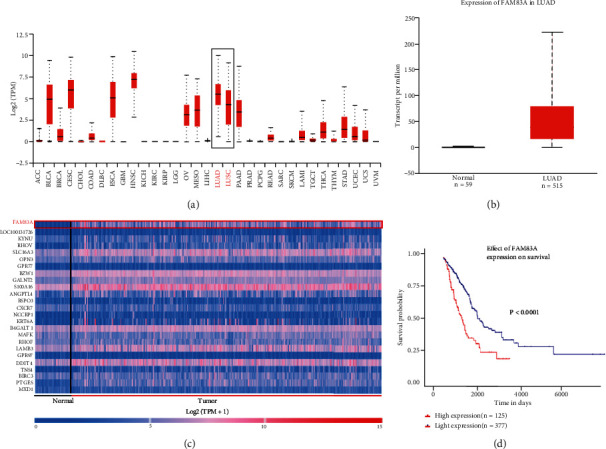
UALCAN analysis of TCGA showed FAM83A is overexpressed in LUAD. (a) UALCAN analysis showed FAM83A is overexpressed in lung adenocarcinoma (LUAD) and lung squamous carcinoma (LUSC), especially the former. (b, c) Cluster analysis showed a significant over expression of FAM83A in LUAD (tumor) than in normal lung tissue (normal). (d) Kaplan-Meier analysis showed that patients with high FAM83A expression had shorter overall survival, *P* < 0.0001.

UALCAN analysis of TCGA (http://ualcan.path.uab.edu/analysis.html) showed that overexpression of FAM83A occurred in NSCLC, especially LUAD (Figure 1(a)). The cluster analysis of the LUAD gene showed a significant increase in transcriptional expression of FAM83A between LUAD and the normal lung tissues (Figures 1(b) and 1(c)). Furthermore, the Kaplan-Meier analysis reported that patients with high FAM83A expression had shorter overall survival (*P* < 0.0001), as shown in Figure 1(d). In conclusion, the obtained results of the TCGA database demonstrated that FAM83A overexpressed in LUAD and closely correlated with a worse prognosis.

### 3.2. High Expression of FAM83A in LUAD is Related to Advanced Clinical and Pathological Characteristics

**Figure 2:**
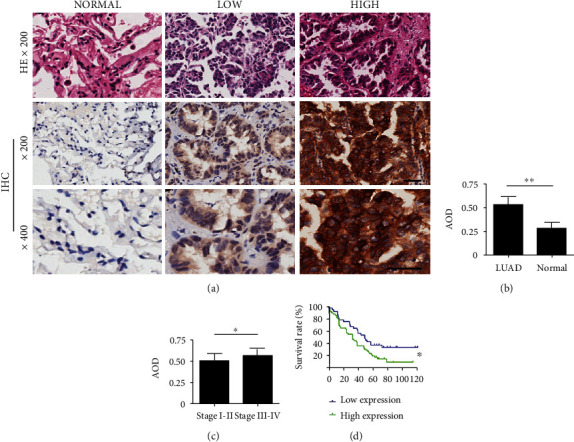
FAM83A protein was overexpressed in LUAD and higher FAM83A expressions related to poor overall patient survival. (a) Representative hematein-eosin (HE) and immunohistochemistry (IHC) images of FAM83A expressions in adjacent normal tissues (NORMAL) and LUAD with low (LOW) and high (HIGH) FAM83A expressions. The short bar is equal to 50 microns; the long bar is equal to 100 microns. (b) The average optical density (AOD) of FAM83A in LUAD tissues and adjacent normal lung tissues. (c) AOD of FAM83A in LUAD tissues in stage I-II and stage III-IV. (d) Kaplan-Meier survival analysis showed a significant difference in 84 LUAD patients grouped by low and high FAM83A expression. ^∗^*P* < 0.05, ^∗∗^*P* < 0.01.

Immunohistochemical staining was used to detect FAM83A protein in the LUAD tissue chip. The obtained results indicated that the FAM83A protein was dyed brown and located mostly in the cytoplasm, with a few in the nucleus (Figure 2(a)). Moreover, high expression of FAM83A in LUAD and low expression in the adjacent normal tissue were found (*P* < 0.01, Figure 2(b)). Subgroup analysis showed that stage III-IV patients had higher FAM83A expression than stage I-II patients (*P* < 0.05, Figure 2(c)). Subsequent correlation analysis suggested that FAM83A expression was correlated with clinical stages (*P* = 0.008) and lymph node classifications (*P* = 0.007), but not with T classification (*P* = 0.634) or metastasis classification (*P* = 0.235). Univariate and multivariate Cox regression analyses indicated that FAM83A expression was an independent prognostic factor for survival in patients with LUAD (hazard ratio: 1.745, 1.879, 95% confidential interval: 1.167–2.945, 1.075–3.321, *P* =0.023, 0.020, respectively) (Table 2). Furthermore, the Kaplan-Meier analysis revealed that patients with high FAM83A expression had shorter overall survival (*P* < 0.01, Figure 2(d)). Consequently, the results indicated a high FAM83A expression in LUAD, which was related to advanced clinical and pathological features and poor prognosis.

### 3.3. FAM83A Modulated Proliferation of LUAD Cells

**Figure 3:**
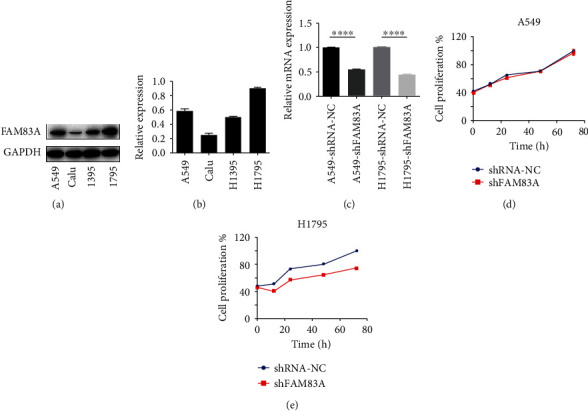
FAM83Apromoted proliferation of LUAD cells. (a, b) Western blotting analysis showed that FAM83A levels were higher in A549 and H1795 cells. (c) The reverse transcription-polymerase chain reaction (RT-PCR) assay demonstrated that A549 and H1795 cells transfected with FAM83A lentivirus (A549/H1795-shFAM83A), showed less FAM83A RNA expressions. (d) The cell proliferation assay showed no differences between A549-shFAM83A and A549-shRNA-NC cells at each time point. (e) The cell proliferation assay showed significant differences between H1795-shFAM83A and H1795-shRNA-NC cells. ^∗∗∗^*P* < 0.001, ^∗∗∗∗^*P* < 0.0001.

To investigate FAM83A expression in cancer cells, its expression in several adenocarcinoma cell lines including A549, H1395, H1795, and Calu-3 cells was detected by Western blotting assay. The results showed that FAM83A expression was higher in A549 and H1795 cells and lower in H1395 and Calu-3 cells (Figures 3(a) and 3(b)). In RT-PCR, A549 and H1795 cells were treated for stable FAM83A-knockdown by shRNA (shFAM83A) and a decrease in FAM83A mRNA levels was observed (Figure 3(c)). A CCK8 cell proliferation assay revealed that FAM83A knockdown could suppress cell proliferation activity in H1795 cells. The proliferation rate of H1795-shFAM83A decreased significantly compared with the control H1795-shRNA-NC cells at each time point (0, 12, 24, 48, and 72 h) (Figure 3(e), *P* < 0.001). In contrast, the suppression was not observed in A549 cells and it was indicated that FAM83A knockdown had no obvious effects on A549 cell proliferation (Figure 3(d), *P* > 0.05).

### 3.4. FAM83A Promoted LUAD Cell Migration Ability

**Figure 4:**
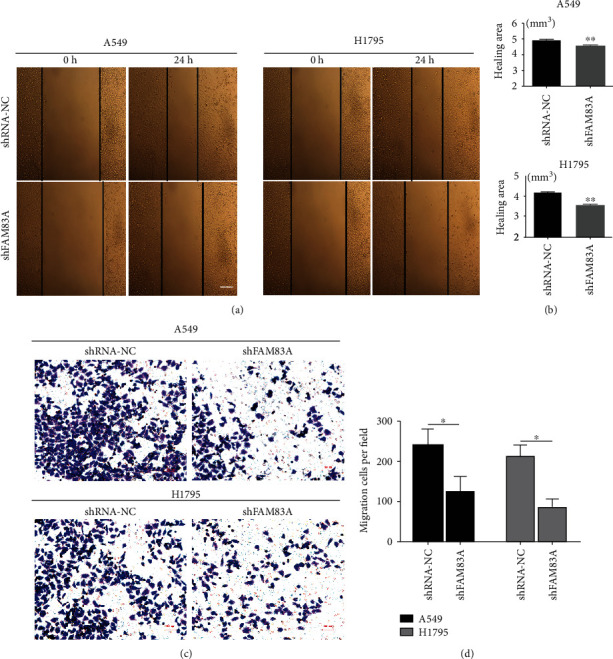
FAM83A promoted LUAD cell migration ability. (a, b) The wound-healing assay showed significantly larger healing area in A549-shFAM83A and H1795-shFAM83A cells. (c, d) The transwell cell migration assay showed that in A549-shFAM83A and H1795-shFAM83A, more cells passed through the basement membrane. ^∗∗^*P* < 0.01, ^∗∗∗^*P* < 0.001.

To investigate the roles of FAM83A in cell migration, wound-healing and transwell cell assays were performed in A549 and H1795 cells. The wound-healing assay showed a significant decrease in the healing area in FAM83A knocked down cells (A549, H1795) (Figures 4(a) and 4(b)), indicating that FAM83A knockdown significantly inhibited the migration ability of A549 and H1795 cells. In addition, FAM83A knockdown in A549 and H1795 cells significantly impaired cell migration ability, as shown in the transwell cell migration assay (Figures 4(c) and 4(d)). More cells passed through the basement membrane of the transwell in FAM83A knocked-down cells, compared with in the controls. The results indicated the promotion of invasion and migration of FAM83A in the lung cancer cell.

### 3.5. FAM83A Induced EMT in LUAD Cells

**Figure 5:**
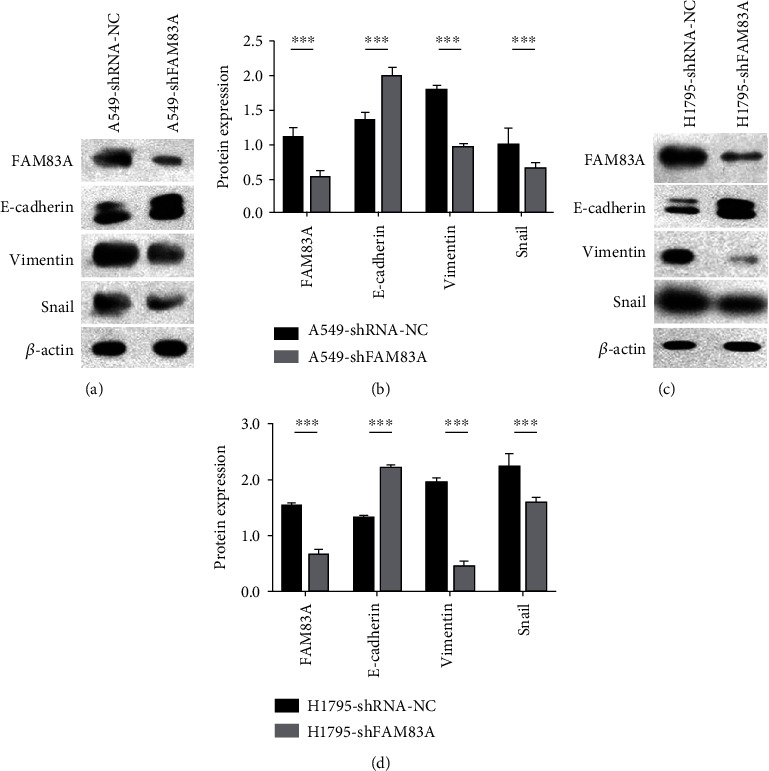
FAM83A induced EMT in LUAD cells. (a, b) Western blotting showed that FAM83A and E-cadherin expression increased, while Vimentin and Snail decreased when FAM83A was knocked down in A549 cells. (c, d) The same results were seen in H1795 cells. *β*-Actin was used as the loading control. ^∗∗∗^*P* < 0.001.

EMT can promote tumor invasion and is a vital step during the early stage of metastasis. In this study, the expression of EMT-related markers was observed in A549 and H1795 cells. FAM83A depletion resulted in upregulation of epithelial marker E-cadherin expression and downregulation of vimentin expression as a mesenchyme marker. Furthermore, the EMT-related transcription factor Snail was downregulated when FAM83A was depleted (Figures 5(a)–5(d)). The results indicated that FAM83A depletion impaired the EMT ability of LUAD cells.

## 4. Discussion

In the present study, high expression of FAM83A was observed in LUAD tissues and it was noticed that FAM83A could affect tumor biological characteristics including cell proliferation, invasion, and metastasis. FAM83A overexpression was associated with advanced clinical and pathological features in LUAD patients. These findings recommended FAM83A as a LUAD oncogene and a potential biomarker for the diagnosis and prognosis of LUAD.

Similar to the results of TCGA database bioinformatics analysis, the previous studies showed an increase in FAM83A expression in LUAD patients [18, 19]. Immunohistochemical staining confirmed that FAM83A expression was increased in LUAD tissue at the protein level. The results allayed the suspicion of whether FAM83A expression was increased at the mRNA and protein levels, as mRNA expression is not always coincident with protein expression [22].

It is well known that FAM83A plays an important role in regulating cell proliferation, differentiation, and invasion. Its role in some tumors has also been well investigated. Lee et al. reported that overexpression of FAM83A in breast cancer promoted cell proliferation and invasion [23]. Chen et al. found that overexpression of FAM83A markedly increased, whereas inhibition of FAM83A decreased cell proliferation in an in-vivo mouse model of pancreatic cancer [24]. The phosphorylation of FAM83A, downstream of EGFR, and upstream of ERK might activate the PI3K/AKT and MAPK signaling pathways, promoting the proliferation, differentiation, apoptosis, and invasion of cells [25, 26]. In the present study, depletion of FAM83A expression inhibited the proliferation, migration, and invasion of lung adenocarcinoma cells. Interestingly, the proliferation capacity of A549 cells exhibited a significant decreasing trend after FAM83A depletion. A possible reason might be a rather limited FAM83A overexpression in A549 cells compared to the H1795 cells. Consequently, the effectiveness of FAM83A knockdown by shRNA remained obscure. A recent study that detected FAM83A expression in nine adenocarcinoma cell lines including A549 cells was detected by the study of Zhou et al. High expression of FAM83A in some cell lines and relatively low expression in A549 cells were observed [27].

EMT is an essential process that promotes adherent epithelial cell movement. EMT can enhance cell mobility and promote tumor progression, and affect cancer features, especially invasion and metastasis [28–31]. In the current study, it was found that FAM83A depletion resulted in the absence of mesenchymal markers, indicating that FAM83A was involved in LUAD EMT processes. In addition, it was observed that under-expression of FAM83A reduced Snail expression, suggesting that FAM83A might regulate the EMT phenotype by inhibiting Snail expressions. Previous studies have shown that constitutive activation of the PI3K/AKT signaling cascade was closely correlated with Snail upregulation and diverse tumor cell metastasis [32–37]. Therefore, investigating whether FAM83A activates Snail and regulates EMT through the PI3K/AKT pathway is worth further studies.

The clinical significance of FAM83A in some cancer types such as breast cancer has been well studied [23]. There is convincing evidence that FAM83A is also related to the prognosis of lung cancer [15]. In the present study, the bioinformatics analysis demonstrated that overexpression of FAM83A was correlated with poor patient survival. Furthermore, immunohistochemical experiments revealed an increase in staining of FAM83A expression in stage III and IV patients, where FAM83A overexpression was positively associated with disease stage and lymph node classification. The results indicated that high FAM83A expression was related to advanced clinical and pathological LUAD characteristics. In breast cancer, FAM83A regulates the proliferation and invasion of cancer cells through the PI3K/AKT pathway. Inhibition of related kinases on this pathway can block the regulator effects of FAM83A on breast cancer [23, 37].

In the present study, the molecular mechanism of FAM83A in the regulation of proliferation, invasion, and EMT was evaluated. It is not clear whether the PI3K/AKT pathways are similarly involved in the regulatory mechanism of FAM83A in LUAD. As well as further studies are required to identify novel drug targets which may provide new therapeutic targets for LUAD. Therefore, it is suggested that these factors must be investigated and should be considered in future studies.

## 5. Conclusion

According to the results, at advanced stages of cancer, transcriptional expression as well as the protein level of FAM83A is increased in lung adenocarcinoma. Due to depletion of FAM83A, there is an increased expression of various types of markers, i.e., epithelial and mesenchyme. This overexpression shown the poor prognosis of FAM83A in lung cancer. In the future, FAM83A might be a potential new target for molecular targeted therapy of patients to its strong association with prognosis and expression of the disease.

## Figures and Tables

**Table 1:** Correlations between FAM83A expression and clinical/pathological characteristics in LUAD.

Characteristics	FAM83A		*P*-value
	Low	High	
Age (y)			0.547
≥60	13	20	
<60	23	28	
Gender			0.784
Male	19	27	
Female	17	21	
T classification			0.634
T1	8	8	
T2	21	26	
T3	6	10	
T4	1	4	
N classification			0.007
N0	22	13	
N1	8	17	
N2	4	15	
N3	2	3	
M classification			0.235
M0	35	44	
M1	1	4	
Clinical stage			0.008
I	17	8	
II	10	12	
III	8	24	
IV	1	4	

**Table 2:** Univariate and multivariate analyses for over survival in patients with LUAD.

Characteristics	Univariate analysis			Multivariate analysis		
	HR	95% CI	*P*-value	HR	95% CI	*P*-value
FAM83A	1.745	1.167-2.945	0.023	1.879	1.075-3.321	0.020

HR: hazard ratio; CI: confidence interval.

## Data Availability

The datasets used and/or analyzed during the current study available from the corresponding author on reasonable request.
